# Discovered and disappearing? Conservation genetics of a recently named Australian carnivorous marsupial

**DOI:** 10.1002/ece3.4376

**Published:** 2018-08-29

**Authors:** Thomas Y. Mutton, Susan J. Fuller, David Tucker, Andrew M. Baker

**Affiliations:** ^1^ School of Earth, Environmental and Biological Sciences, Science and Engineering Faculty Queensland University of Technology Brisbane QLD Australia; ^2^ Natural Environments Program South Brisbane QLD Australia

**Keywords:** *Antechinus mysticus*, *Antechinus subtropicus*, connectivity, conservation, dasyurid, population genetics

## Abstract

Five new species within the Australian carnivorous marsupial genus *Antechinus* have recently been named, at least two of which are threatened. Important facets of the habitat use and extinction risk of one of these new species, the buff‐footed antechinus, *A*.* mysticus*, are not well understood. Previous research has suggested that the species utilizes a broad range of inter‐connected forest habitats in southeast Queensland (Qld), Australia. Based on this potentially connected habitat, we predicted that *A. mysticus* should have low population genetic structure, particularly in relation to its congener, the spatially restricted, high altitude, closed‐forest *A. subtropicus*. We genotyped nine microsatellite loci for six populations of *A*.* mysticus,* sampled throughout their known range in eastern Australia, and compared them with four proximate populations of *A*.* subtropicus*. Surprisingly, genetic structuring among southeast Qld populations of *A*.* mysticus* was moderate to high and similar to that between *A*.* subtropicus* populations. We postulate that all *A. mysticus* populations have declined recently (<100 generations), particularly the northernmost southeast Qld population, which may be at risk of extinction. Our results suggest that *A*.* mysticus* is limited to a more scattered and fragmented distribution than previously thought and may be in decline. The identification of population decline in this study and recently in other *Antechinus* suggests the extinction risk of many Australian mammals should be reassessed.

## 1 INTRODUCTION

Differences in population genetic patterns between species with similar ranges and life histories can reveal previously overlooked facets of a species’ ecology or biogeography. The range and historical connectivity of a species are often assumed on the basis of the habitat types in which they have been found. In closely related species, which are believed to occupy largely separate habitats across a similar range, a comparative analysis of population genetics allows the formulation and testing of assumptions about differential habitat use, past connectivity, and biogeography (Avise, 2000; Manel, Schwartz, Luikart, & Taberlet, 2003). Understanding these patterns is integral to effective conservation management of a species, as are the estimates of genetic diversity such studies provide.

*Antechinus* are one of only a few mammal genera worldwide which exhibit semelparous reproduction and, as a consequence, have been widely studied as a model in breeding biology (Naylor, Richardson, & Mcallan, 2008). Such interest has also encouraged a number of genetic studies on *Antechinus*, with a principal focus on deeper level systematics (Mitchell et al., 2014; Westerman et al., 2015), taxonomy (Baker, Mutton, Mason, & Gray, 2015; Van Dyck, 2002) and a range of single population studies mainly focusing on habitat use (Banks et al., 2005; Kraaijeveld‐Smit, Lindenmayer, Taylor, Macgregor, & Wertheim, 2007; Lada, Thomson, Mac Nally, & Taylor, 2008). However, genetic studies of the genus have generally not been undertaken across a species’ total geographic range. Such a comprehensive evaluation may provide invaluable information on the life history, habitat use, and extinction risk of a species. Recently, five new *Antechinus* species have been named based on combined phylogenetic, morphology and breeding biology data, and at least two of these are at risk of extinction (Baker, Mutton, & Hines, 2013; Baker, Mutton, Hines, & Van Dyck, 2014; Baker, Mutton, & Van Dyck, 2012; Baker et al., 2015). Currently, little is known about the ecology or population genetics of these five species. Consequently, there is a vital need for fine‐scale, yet geographically detailed molecular studies on the suite of newly discovered species. Understanding species’ ranges, population structure, demography, and habitat preference is crucial for effective conservation and is particularly important in Australia where mammals have suffered an extremely high rate of extinction in the last 200 years (Woinarski, Burbidge, & Harrison, 2015).

The buff‐footed antechinus*, A*.* mysticus*, was discovered in 2012 (Baker et al., 2012). *Antechinus mysticus* and its congener, the subtropical antechinus*, A*.* subtropicus,* share a similar range in southeast Queensland (Qld) but occupy largely separate habitats (Baker et al., 2012; Mutton, Gray, Fuller, & Baker, 2017; Pearce, 2016). *Antechinus subtropicus* inhabits high altitude subtropical vine forest and rainforest patches scattered throughout the protected habitats of near coastal southeast Qld (Baker et al., 2012; Van Dyck, Gynther, & Baker, 2013). *Antechinus mysticus* occupies comparatively drier, more open and lower altitude environments of southeast Qld, except for the single known population in mid‐east Qld which occurs in rainforest (Baker et al., 2012; Mutton et al., 2017; Pearce, Burwell, & Baker, 2017).

Based on the fragmented nature of *A. subtropicus*’ habitat, and the potential connectivity of *A. mysticus*’ habitats, we expected that the relative connectivity of the two species to be reflected in differing population genetic signatures. The lower altitude, drier forest habitats of *A*.* mysticus* would presumably have been more connected in recent geological time than the comparative “islands” of high altitude vine forest and rainforest which *A*.* subtropicus* favor (Byrne et al., 2011). Over time, such geographic partitioning could lead to a relatively higher degree of genetic structuring among *A. subtropicus* populations in southeast Qld. Mitochondrial cytochrome b (Cytb) results support this contention, with *A. mysticus* showing very little diversity in southeast Qld, comprising just two haplotypes, only one base pair different (0.2%), across its range (Baker et al., 2012; Mutton, 2017). In contrast, *A. subtropicus* showed four times as much genetic divergence (0.8%) over only half the geographic distance (75 km; Mutton, 2017) and greater divergence in Cytb has been recorded within antechinus populations and across similar geographic distances in a number of species (Beckman, Banks, Sunnucks, Lill, & Taylor, 2007; Mutton, 2017). The two species, which are not closely related within the genus, show a deep interspecies divergence (13.3%–14.3% at Cytb ‐ Baker et al. (2012)). The differing habitat preference of these species also appears to hold in known areas of sympatry. The two species can co‐occur at mid‐altitude (~250 m) and transitional environments/ecotones where open forest and closed, vine forest, and rainforest communities merge, but each occur in the absence of congeners in higher altitude rainforest (*A. subtropicus*) and lower altitude open forest (*A. mysticus*) of southeast Qld (Baker et al., 2012; Mutton et al., 2017).

To test the hypothesis of greater population connectivity in *A*.* mysticus* than *A*.* subtropicus,* we sampled the majority of known populations of *A. mysticus* and representative populations throughout the range of *A. subtropicus*. We compared the species’ population structures using nine microsatellite loci and examined levels of genetic diversity within and between populations, which is essential for effective conservation management. Microsatellites evolve more rapidly than mitochondrial markers, allowing more recent divergence and finer scale genetic structuring to be revealed. Mitochondrial genes can also be biased by sex‐specific dispersal patterns, as they are maternally inherited. In contrast, microsatellites are inherited from both parents and population size and stochastic factors can have a significant influence on genetic estimates (Selkoe & Toonen, 2006). These attributes make microsatellites an ideal marker to test the ideas posed in this study.

## 2 MATERIALS AND METHODS

### 2.1 Study sites

#### 2.1.1 *A. mysticus*

**Figure 1:**
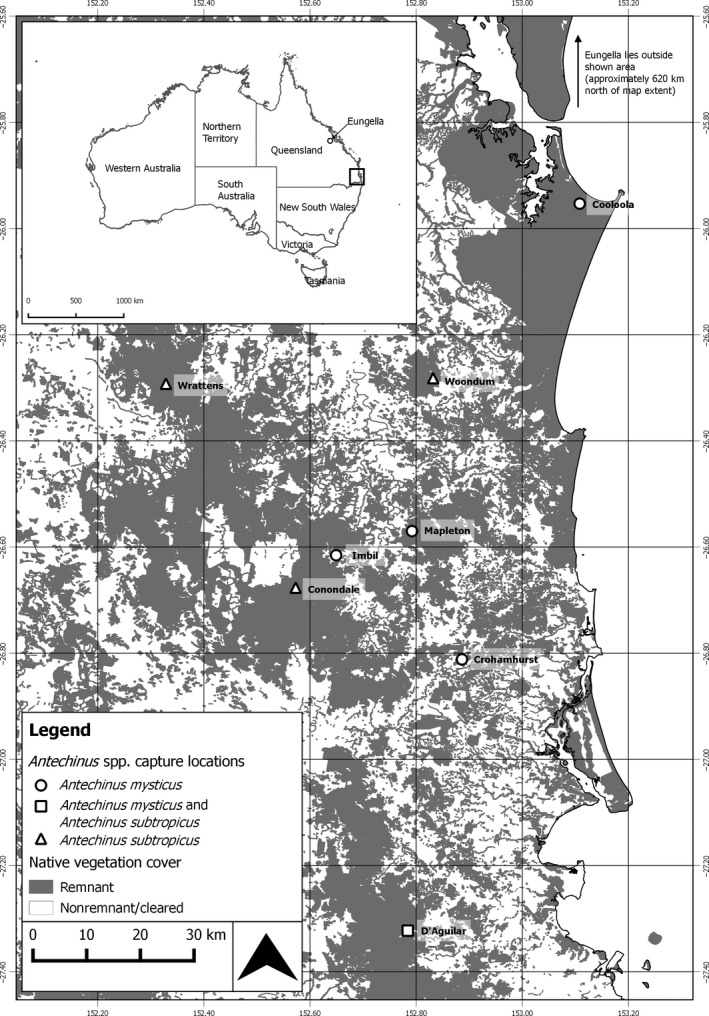
Map of southeast Queensland showing remnant vegetation and the *Antechinus* sites sampled in the present study. Circles represent *A*.* mysticus* populations, triangles represent *A*.* subtropicus* populations. The square represents a site at which both species were caught

Samples were collected from across the known geographic range of *A*.* mysticus*, including five localities in southeast Qld and one from Eungella, mid‐east Qld (Figure 1) (Baker et al., 2012; Mutton et al., 2017). Despite extensive trapping effort (cumulatively more than 10,000 trap nights across eight locations), *A*.* mysticus* has not been found in the approximately 700 km straight line distance (SLD) separating southeast Qld from Eungella or from other localities in mid‐east Qld (Baker et al., 2012, 2013; Mutton et al., 2017). In southeast Qld, our sampled *A*.* mysticus* sites ranged from 15 to 155 km SLD apart and 130 to 230 m above sea level (ASL) (Appendix S1 and S2). Eungella is located 709‐810 km SLD from the other sites and at a much higher altitude (750 m ASL) than other *A. mysticus* populations (Appendix S1 and S2). Study sites were situated in areas of protected remnant vegetation (Figure 1). The southeast Qld *A*. *mysticus* sites were predominantly riparian open woodland, while the mid‐east Qld site was dominated by rainforest (Pearce, 2016) (See Appendix S3 for vegetation community information). D'Aguilar was the only site surveyed (and known) at which both species occur (Figure 1). This site is typified by an abrupt merging of riparian open woodland and rainforest communities (Mutton et al., 2017).

#### 2.1.2 *A. subtropicus*

*Antechinus subtropicus* samples were collected from four sites covering the entire known range of the species, except the southern latitudinal maximum at Border Ranges (Figure 1). Despite numerous surveys over a 3‐year period, including more than 5,000 cumulative trap nights at several locations, we were unable to trap *A. subtropicus* at this location (Mutton, 2017). Three of our *A*.* subtropicus* sites were approximately equidistant (~50 km SLD) (Wrattens, Woondum and Conondale), while the fourth site, D'Aguilar, was located 75–124 km SLD south of these sites (Figure 1). The sites were located within patches of remnant vegetation (Figure 1). Excluding D'Aguilar, where the two species co‐occur, all *A*.* subtropicus* sites were high altitude, ranging from 460‐ to 708 m ASL (Appendix S1 and S2). The sites were dominated by *Eucalyptus* species and notophyll vine forest (Appendix S3).

#### 2.1.3 Mapping

Southeast Qld study site locations were mapped in relation to remnant vegetation cover (DEHP, Queensland Government 2009) and elevation (Geoscience Australia 2011) using QGIS software V 2.12.0 (QGIS Development Team 2017). Remnant vegetation cover described native vegetation that had not been cleared or had been cleared but retained a dominant canopy >70% of the height and >50% of the cover relative to the undisturbed height and cover of that layer and was dominated by species characteristic of the vegetation's undisturbed canopy (DEHP, Queensland Government 2017). Elevation data were generated using a 1‐s SRTM Derived Digital Elevation Model. Elevation was categorized and color‐coded into 175 m increments between 0 m and ≥700 m.

### 2.2 Taxon sampling

Species were identified by eye based on pelage color as described in Baker et al. (2012), and their identity confirmed using mtDNA (Cytb) sequencing (Mutton, 2017), prior to microsatellite screening. Genetic samples were obtained through field collection with type A Elliott traps (Elliott Scientific, Vic, Australia). Ear tissue samples were collected from individuals captured in the field and stored in 80% ethanol. Samples from Eungella and D'Aguilar were collected during parallel capture–mark–recapture studies described in Pearce (2016) and Mutton et al. (2017), respectively. Cooloola samples were collected over a number of months in approximately 9,000 trap nights. Samples from the other sites were collected by trapping for 3–5 days and between 500 and 1,000 trap nights at each site during the winter prebreeding period.

Nine microsatellite loci (see Table 1) were amplified for both species using a Qiagen multiplex kit (Qiagen, Dusseldorf, Germany). All primers were originally designed for *A. agilis,* except one (Aa7Q), which was designed for *A. flavipes* (see Table 1). As allele sizes of some loci overlapped, two multiplexes were amplified separately (Table 1). For each multiplex, an individual primer mix was made containing 4 μl of each forward and reverse primer (100 pmol) with diluted water added to a final volume of 200 μl. Microsatellite fragments were amplified in a PCR reaction containing 1 μL of 1:10 diluted gDNA, 4 μl of RNase‐free H_2_O, 1.4 μl of primer mix and 6.25 μl of 1× Qiagen multiplex master mix. The following PCR cycle protocol was used for both microsatellite groupings: 95°C for 5 min; 30 cycles of 94°C for 30 s, 53°C for 90 s, and 72°C for 30 s; with a final extension at 72°C for 10 min. Fragments were analyzed on an ABI 3500 sequencing platform in a sequencing reaction containing: 10 μl of Hi‐Di^™^ formamide (Applied Biosystems, Carlsbad, California, USA), 1 μl of GSLIZ600 sequencing size standard (Applied Biosystems), and 1 μl of a 1/3 dilution of each PCR product.

**Table 1:** Primer sequence and references of the nine microsatellites loci genotyped in this study. The primers were divided into two multiplex groupings

Locus	Primer sequence	Reference	Multiplex
Aa1A	F (5′‐TCAGCCTCGATATTTTTCTAATG‐3′)	Banks et al. (2005)	A
	R (5′‐AGCTCCTTTTGTATCCTAAC‐3′)		
Aa4A	F (5′‐TTTGATCCTCAGAGACTTGAT‐3′)	Banks et al. (2005)	A
	R (5′‐CCAAATCTACGTAAAATATCC‐3′)		
Aa4K	F (5′‐TCTGTGGAGCCTCTAGAGAAT‐3′)	Kraaijeveld‐Smit, et al. (2002)	A
	R (5′‐AAGAGGATAACCCATTCAGA‐3′)		
Aa7D	F (5′‐GGATTTGATCTCAGGTTTTC‐3′)	Kraaijeveld‐Smit et al. (2002)	A
	R (5′‐ATATCCACCAATGACTGCAA‐3′)		
Aa7K	F (5′‐TTTCTGGATGAACAGTTTGA‐3′)	Banks et al. (2005)	A
	R (5′‐GAGATGTGAGCAGTTAGTGGAC‐3′)		
Aa7H	F (5′‐AATTCAGTTGAGTCCACTTTG‐3′)	Banks et al. (2005)	B
	R (5′‐GTGCTTTCTCTGTCTTTCC‐3′)		
Aa7M	F (5′‐TGCTTTGTTCTTGCTAAGTA‐3′)	Banks et al. (2005)	B
	R (5′‐ACAATCATATGTTTATGTAGCC‐3′)		
Aa7O	F (5′‐GTCTTTGGATAATTGAAGTCTG‐3′)	Kraaijeveld‐Smit et al. (2002)	B
	R (5′‐GAATGAGGATCTAAGTGAATGT‐3′)		
Aa7Q	F (5′‐AAGCCCTGACAAATGGT‐3′)	Lada, et al. (2007)	B
	R (5′‐ATTCACTGTGCCATCAACTACCT‐3′)		

### 2.3 Genetic variation

Allele size was scored in GeneMapper 4.1 (Applied Biosystems). The total number of alleles (*A*), private (unique) alleles (*uA*), rare alleles (*rA;* frequency ≤5%), observed (*H*_o_), and expected (*H*_e_) heterozygosity per population were estimated using GenAlEx 6.502 (Peakall & Smouse, 2006). Allelic richness was standardized for sample size (10 individuals) (*AR*) and estimated in Fstat 2.9.3.2 (Goudet, 1995). As one locus (Aa70) had low sample sizes at two *A. subtropicus* sites, it was excluded from *AR* analysis for this species. Wilcoxon sign‐rank tests were implemented in R to compare differences in *AR*,* rA,* and *H*_e_ between populations of each species.

Tests for linkage disequilibrium and departures from Hardy–Weinberg Equilibrium (HWE) for each locus‐population combination were carried out in Genepop 4.2 (Rousset, 2008) using 1,000 permutations to test for statistical significance. To assess whether sample effort was adequate to capture the allelic diversity of each population, we undertook rarefaction analysis using the “jackmsatpop” function of the R package PopGenKit 1.0 (Paquette, 2012). Rarefaction curves were generated by calculating the sample size adjusted allelic diversity at stepwise increases of one until the sample size of each population was reached. Two hundred jackknife replicates were performed at each interval.

### 2.4 Population structure and genetic bottlenecks

Genetic structure between populations was analyzed using pairwise *F*_ST_ (Weir & Cockerham, 1984). As all measures of population differentiation have limitations, it has been suggested that multiple population differentiation statistics be examined (Meirmans & Hedrick, 2011)_._ Therefore, we also calculated *D*_EST_ in GenAlEx 6.502 (Jost, 2008). Hierarchical structuring of populations was assessed by an Analysis of Molecular Variance (AMOVA; Excoffier, Smouse, & Quattro, 1992). The relationship between geographic distance and genetic distance (pairwise *F*_ST_) was analyzed using Mantel tests (Mantel, 1967). Pairwise *F*_ST_, AMOVA, and Mantel tests were undertaken in Arlequin 3.5.2.2 (Excoffier & Lischer, 2010). The pairwise *F*_ST_ critical value (*α*) was corrected for multiple tests using the BY False Discovery Rate method, which accounts for Type I error with less loss of power than the Bonferroni adjustment (Narum, 2006).

We also conducted redundancy analysis (RDA), which allowed us to compare with Mantel tests, and more accurately calculates isolation by distance and the influence of geographic variables on genetic structure (Legendre & Fortin, 2010; Meirmans, 2015). RDA was undertaken using the R package Vegan 2.4‐5 (Oksanen et al., 2017; R Core Team, 2015). We used allele frequencies as the dependent variable and four independent variables: latitude, longitude, degree of isolation (IS), and minimum distance to neighboring site (DN). IS was measured as the mean distance to the closest three sites. Stepwise AIC comparisons (using the function “ordistep”) of the full and nested RDA models were undertaken to determine the optimal model.

Genetic evidence for a recent (2–4 *Ne* generation) reduction in population size was tested using BOTTLENECK 1.2.02 (Piry, Luikart, & Cornuet, 1999). A two‐phased mutation model (TPM) incorporating 95% single‐step changes, 5% multi‐step changes with a variance among multiple‐step changes set to 12% was used (Piry et al., 1999). Following the recommendations of Piry et al. (1999), statistical significance was evaluated from 5,000 simulations of the one‐tailed Wilcoxon sign‐rank test. For each population, Garza and Williamson's (2001) M‐ratio was calculated using Arlequin 3.5.2.2. M‐ratio is a measure of the proportion of unoccupied allelic states given the allele size range. It is sensitive to population bottlenecks (often for over 100 generations) as the ratio reduces when alleles are lost due to random drift after a bottleneck occurs (Garza & Williamson, 2001). The inbreeding coefficient (*F*_IS_) was estimated for all loci in each population using Arlequin 3.5.2.2 (Excoffier & Lischer, 2010), with 10,000 permutations of alleles among individuals within a population to test for significance.

To define the number of distinct population groups (*K*) within each species, Bayesian clustering of individuals without prior assignment to population was performed using the software package STRUCTURE 2.3.4 (Pritchard, Stephens, & Donnelly, 2000). The program was run for 10 iterations of 100,000 generations, with an initial burn‐in of 20,000 generations. The value of *K* was set from 1 to 10, with ten replicates of each *K* to verify the convergence of the Markov chain. No assumptions were made about the shared descent of populations, allele frequencies were set to uncorrelated, and separate alpha values were used for each population (Pritchard et al., 2000). The program assigns individuals to *K* clusters, with the user nominating which value of *K* is most appropriate for their data. Evanno, Regnaut, and Goudet (2005) recommend that the highest value of ∆*K* be taken as the “true” *K* value. However, it has also been suggested that the value of *K* which captures the majority of structure in the dataset be used (Pritchard et al., 2000), a technique which has been implemented in a number of ecological studies (Bryant & Fuller, 2014; Cardoso, Mooney, Eldridge, Firestone, & Sherwin, 2014; Krosch et al., 2013). Thus, ∆*K* values were plotted and STRUCTURE results were summarized using Structure Harvester (Earl & Vonholdt, 2012). Cluster membership coefficient matrices for each *K* value were summarized using *CLUMPP* 1.1.2 (Jakobsson & Rosenberg, 2007) and visualized as admixture graphs with *Distruct* 1.1 (Rosenberg, 2004).

## 3 RESULTS

### 3.1 Descriptive statistics

In total, 101 *A*.* mysticus* (39% female) and 75 *A*. *subtropicus* (31% female) individuals were screened for nine microsatellite loci. One locus, Aa7Q, failed to amplify for all *A*.* mysticus,* presumably due to a mutation in the primer binding region (Selkoe & Toonen, 2006). For *A*.* mysticus* no site significantly departed from HWE for more than two of the eight loci, but one locus (Aa7K) significantly departed from HWE at four of the six sites. This suggests that null alleles are present or the locus is under selection (Selkoe & Toonen, 2006). We therefore excluded this locus from all further analyses for *A. mysticus*. In contrast, no locus significantly departed from HWE for more than two sites for *A*.* subtropicus*, but at one site (D'Aguilar), six of the nine loci significantly departed from HWE. Patterns of genetic diversity and structure were initially tested using the seven loci successfully genotyped for both *A. mysticus* and *A. subtropicus*. However, a comparison of results for *A. subtropicus* showed that there was little difference between the seven and nine loci datasets, and therefore, the final analysis included seven loci for *A. mysticus* and nine for *A. subtropicus*.

Pairwise comparison between all individual loci revealed low rates of significant linkage disequilibrium for *A*.* mysticus* (4.8%) and *A*.* subtropicus* (4.0%). As there was no consistent pattern of linkage across sites or loci (data not shown), it was deemed unlikely that these low rates of linkage disequilibrium were due to physical linkage (Selkoe & Toonen, 2006).

Number of samples genotyped per site varied from 12 to 27 for *A*.* mysticus* and 16 to 23 for *A*.* subtropicus* (Table 2). The low number of samples genotyped at some sites and the small number of loci analyzed may limit statistical power (Putman & Carbone, 2014). However, rarefaction analyses suggested sample sizes were adequate to capture most of the genetic diversity at all sites, except for *A. mysticus* at Imbil, where a plateau was not achieved (data not shown). An approximately equal number of alleles were observed for *A*.* mysticus* (214) and *A*.* subtropicus* (215), and the range of alleles per locus was the same for both species (2–9). The average *A* per site was also similar for both species (5.2, *A. mysticus*; 6.0, *A*.* subtropicus*), ranging from 3.4 (Cooloola) to 6.3 (D'Aguilar) for *A*.* mysticus* and 4.8 (Wrattens) to 6.8 (D'Aguilar) for *A*.* subtropicus*. *AR* and *H*_e_ were significantly lower at Cooloola than at any other *A. mysticus* population (*p* < 0.05), except Eungella where there was not a significant difference in *H*_e_ between populations (*p* = 0.06; see Table 2). For *A. subtropicus*, there was a significant difference in *AR* between the Wrattens (4.84) and Woondum (5.94) sites (*p* = 0.02; Table 2). *rA* was significantly lower at Wrattens (0.22) in comparison to D'Aguilar (2.0; *p* = 0.02) (Table 2). There was not a significant difference in *AR, H*_e_ *,* and *rA* between the two species (*p* > 0.5). Private alleles were low at all sites for both species (Table 2).

**Table 2:** Summary of genetic variation in sampled populations of (a) *A*.* mysticus* and (b) *A*.* subtropicus* based on seven and nine amplified microsatellite loci, respectively

Site	*N*	*A*	*AR*	*uA*	*rA*	*H*_e_	*H*_o_	*F*_IS_	M‐ratio
(a)									
Eungella*	13	38	5.23 ± 0.75	0.86 ± 0.46	0.71 ± 0.33	0.68 ± 0.08	0.75 ± 0.10	−0.113	0.51
Cooloola	27	24	3.03 ± 0.27	0.14 ± 0.14	0.71 ± 0.08	0.48 ± 0.08	0.52 ± 0.09	−0.075	0.45
Mapleton	13	35	4.75 ± 0.29	0	1.14 ± 0.03	0.67 ± 0.04	0.65 ± 0.05	0.021	0.48
Imbil	13	38	5.22 ± 0.50	0.14 ± 0.14	1.14 ± 0.12	0.69 ± 0.05	0.61 ± 0.06	0.112	0.48
Crohamhurst	12	38	5.17 ± 0.56	0.29 ± 0.18	1.57 ± 0.31	0.68 ± 0.04	0.71 ± 0.07	−0.037	0.46
D'Aguilar^	23	41	5.25 ± 0.24	0.57 ± 0.30	1.14 ± 0.16	0.75 ± 0.02	0.69 ± 0.05	**0.086**	0.46
(b)									
Wrattens*	17	43	4.84 ± 0.60	0.44 ± 0.34	0.22 ± 0.08	0.69 ± 0.06	0.63 ± 0.10	−0.255	0.495
Woondum	19	56	5.94 ± 0.54	0.67 ± 0.24	1.44 ± 0.29	0.73 ± 0.03	0.67 ± 0.07	**0.502**	0.654
Conondale	16	55	5.94 ± 0.56	0.67 ± 0.17	1.11 ± 0.01	0.70 ± 0.04	0.60 ± 0.09	−0.239	0.667
D'Aguilar	23	61	5.43 ± 0.43	1.11 ± 0.35	2.00 ± 0.09	0.69 ± 0.04	0.50 ± 0.04	−0.054	0.736

*Notes*.

*N:* sample size; *A:* total number of alleles; *AR:* allelic richness standardized for allele size; *uA:* unique (private) alleles; *rA:* rare alleles (frequency ≤5%); *H*_e_: expected heterozygosity; *H*_0_: observed heterozygosity; *F*_IS_: inbreeding coefficient.

*F*_IS_ values significantly different (*p* < 0.05) are shown in bold. Populations showing a significant signature of genetic bottleneck or heterozygote deficit are indicated with an asterisk (*) or caret (^), respectively. M‐ratio is the Garza‐Williamson index following Garza and Williamson (2001).

### 3.2 Genetic bottlenecks and inbreeding

BOTTLENECK 1.2.02 detected a significant heterozygote excess at Eungella and a significant heterozygote deficit at D'Aguilar for *A. mysticus*. The latter was also the only *A. mysticus* site in which F_IS_ analysis showed evidence of significant inbreeding (Table 2). Evidence of a significant genetic bottleneck was also found in one *A*.* subtropicus* site (Wrattens), which was also the only *A. subtropicus* site with an M‐ratio substantially below the critical value Garza and Williamson (2001) calculated for wild populations (0.68) (Table 2). F_IS_ analysis returned one population of *A*.* subtropicus* (Woondum) with evidence of significant inbreeding (Table 2). All *A*.* mysticus* sites had M‐ratio values substantially below the critical value.

### 3.3 Spatial patterns

#### 3.3.1 *A*.* mysticus*

Pairwise *F*_ST_ estimates revealed significant structure among all *A*.* mysticus* sites (Table 3). The northernmost southeast Qld site, Cooloola, exhibited greater differentiation (*F*_ST_ =* *0.217–0.339) than the geographically more isolated Eungella site (*F*_ST_ = 0.136–0.339) (Table 3). Differentiation was substantially lower between the other sites (*F*_ST_ =* *0.053–0.113) (Table 3). Pairwise *D*_EST_ estimates revealed a similar structure to *F*_ST_, although values were much higher (see Appendix S4).

**Table 3:** Pairwise *F*_ST_ estimates of (a) *A*.* mysticus* and (b) *A*.* subtropicus* populations for 7 and 9 amplified microsatellite loci, respectively

(a)	Eungella	Cooloola	Mapleton	Imbil	Crohamhurst
Eungella					
Cooloola	0.339				
Mapleton	0.206	0.260			
Imbil	0.194	0.217	0.087		
Crohamhurst	0.172	0.293	0.113	0.069	
D'Aguilar	0.136	0.222	0.091	0.053	0.094

(b)	Wrattens	Woondum	Conondale
Wrattens			
Woondum	0.044		
Conondale	0.096	0.062	
D'Aguilar	0.134	0.090	0.107

Notes.

All pairwise comparisons were significantly differentiated after adjusting the critical value (*α* < 0.05) using the BY False Discovery Rate correction.

AMOVA showed 18.5% of total genetic variation was partitioned among the six *A*.* mysticus* sites (*p* < 0.001). If the Eungella site was excluded, the total genetic variation between sites was slightly lower (17.0%; *p* < 0.001). However, if the Cooloola site was excluded, total variation among sites was reduced by ~36% (11.8%; *p* < 0.001). When Eungella and Cooloola were both excluded, total genetic variation among the four southeast Qld *A*.* mysticus* sites was 8.4% (*p* < 0.001). Mantel tests did not indicate a significant relationship between genetic and geographic distance when all sites were included (*R* ^xy^ = 0.354, *p* = 0.233), or when the strongly genetically differentiated Eungella (*R* ^xy^ = 0.581, *p* = 0.187) or Cooloola samples were individually excluded (*R* ^xy^ = 0.380, *p* = 0.219), or when both these sites were excluded together (*R* ^xy^ = −0.364, *p* = 0.751).

**Figure 2:**
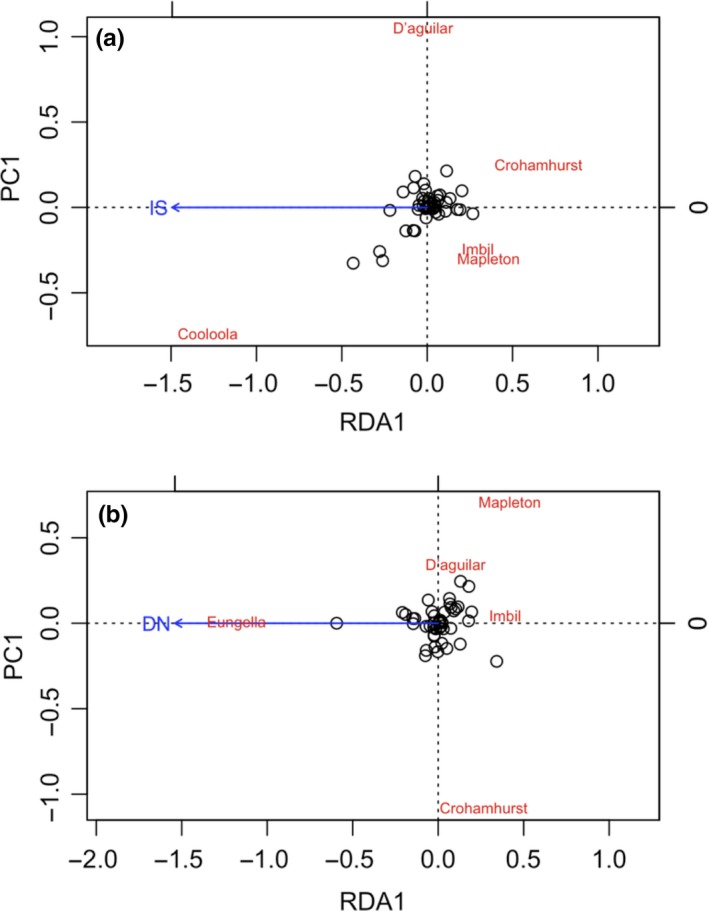
RDAs showing the contribution of spatial factors to genetic structure in *A. mysticus*, optimal models are shown for when (a) Eungella and (b) Cooloola sites were respectively excluded from the analysis. Each site is shown in RDA space and circles show how explanatory variables fall in the RDA space. IS: degree of isolation and DN: distance to nearest neighbour

Stepwise selection revealed that no geographic variables significantly explained genetic variation when RDA included all populations, or when the divergent Eungella and Cooloola populations were both excluded. However, when only the Eungella population was excluded from the analysis, the optimal model included degree of isolation (IS) as an explanatory variable (*p* = 0.025) (Figure 2a). This model explained 38.8% of variance. When the Cooloola population was excluded, distance to nearest neighbor (DN) was revealed as a significant explanatory factor (*p* = 0.016), and the model containing this factor explained 47.1% of observed variation (Figure 2b).

**Figure 3:**
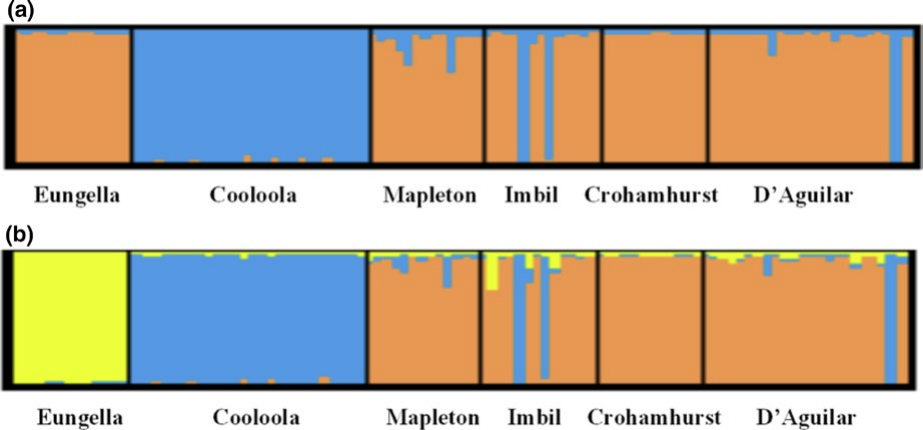
Graphical representation of membership coefficients of the Bayesian STRUCTURE analysis of 7 microsatellite loci for *A*.* mysticus* obtained from 6 sites across the known range of the species. Each plot represents different population assignments for *K*: (a) *K *=* *2; and (b) *K *=* *3. Solid black lines delineate the 6 different sites; each vertical line represents a single individual. Colors represent cluster assignments

Bayesian clustering analysis revealed a pattern of genetic structure concordant with the *F*_ST_ analysis. The Evanno et al. (2005) method identified two groups (*K *=* *2) (Appendix S5a). Graphical representation showed one group to largely correspond to Cooloola and the other group to contain all other sites (Figure 3a). However, exploration of additional groupings revealed further population structuring. The geographically isolated Eungella site was revealed at *K* = 3 as a separate cluster (Figure 3b). Cooloola represented a second cluster which showed a small amount of admixture with the third grouping, which encompassed the other southeast Qld sites (Figure 3b). Increasing *K* to higher values increased admixture and did not reveal further biologically informative groupings (data not shown).

#### 3.3.2 *A*.* subtropicus*

Of the three equidistant (~50 km) sites, Conondale was more divergent from the other two sites (*F*_ST_ = 0.062–0.096) than Woondum and Wrattens were from each other (*F*_ST_ = 0.044) (Table 3). The more geographically separate D'Aguilar site (75–123 km SLD from other sites) was the most genetically differentiated (*F*_ST_ = 0.09–0.134) (Table 3). A similar pattern was revealed in the pairwise *D*_EST_ estimates, although values were much larger (see Appendix S4).

AMOVA revealed 8.93% of total genetic variation partitioned among the four *A*.* subtropicus* sites (*p* < 0.001) and pairwise *F*_ST_ estimates showed a significant difference between all *A*.* subtropicus* sites (pairwise *F*_ST_ = 0.044–0.134) (Table 3). Mantel tests did not show a significant effect of isolation by distance between the *A*.* subtropicus* sites (*R* ^xy^ = 0.695, *p* = 0.121). Stepwise selection revealed that no geographic factors significantly (*p* < 0.05) explained genetic variation.

**Figure 4:**
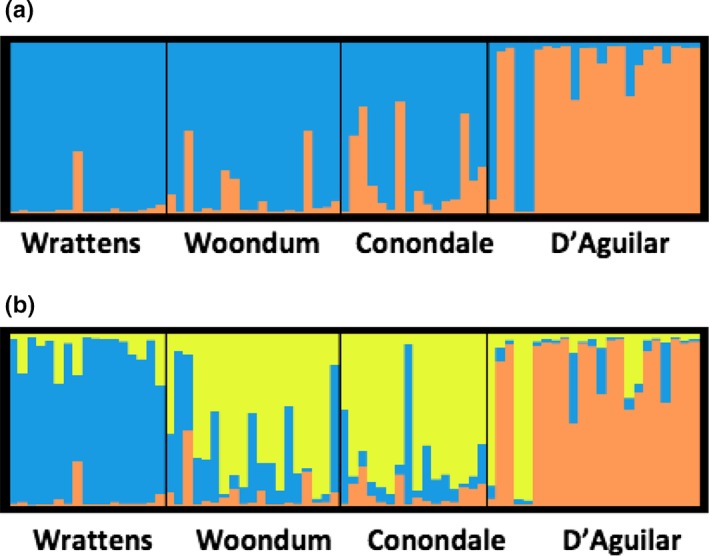
Graphical representation of membership coefficients of the Bayesian STRUCTURE analysis of 9 microsatellite loci for *A*.* subtropicus* obtained from 4 sites in SE Qld. Each plot represents different population assignments for *K*: (a) *K *=* *2; (b) *K *=* *3. Solid black lines delineate the 4 different sites; each vertical line represents a single individual. Colors represent cluster assignment

Bayesian clustering analysis also revealed D'Aguilar as the most differentiated population, with the Evanno et al. (2005) method revealing two groupings (*K* = 2), one of which largely corresponded to D'Aguilar (Figure 4a, Appendix S5b). Admixture between D'Aguilar and the other sites decreased as geographic distance increased (Figure 4a). As for *A*.* mysticus*, the Evanno et al. (2005) method may have under‐represented population structure in *A*.* subtropicus*. Graphical representation of *K* = 3 also revealed D'Aguilar as a largely discrete grouping, but additionally showed Wrattens to be separate from the Woondum/Conondale sites, which formed a third grouping (Figure 4b).

## 4 DISCUSSION

Microsatellite genotyping indicated limited gene flow among populations of *A. mysticus* across its range. This is contrary to our expectations based on ecology, distribution, habitat preference, and mitochondrial gene sequencing of this species (Baker et al., 2012; Mutton, 2017). Indeed, the degree of population genetic structuring appears similar to that observed among populations of scattered high altitude, closed‐forest *A. subtropicus*.

### 4.1 Genetic differentiation and variation between *A. mysticus*,* A. subtropicus*, and other carnivorous marsupials

Two highly differentiated *A. mysticus* populations (Eungella and Cooloola) were revealed in this study. As heterozygosity and allele frequency distribution strongly influence *F*_ST_ results, comparisons of *F*_ST_ values between taxa need to be made with caution (Jakobsson, Edge, & Rosenberg, 2013). However, a general comparison of trends can be revealing. It is notable that the differentiation between each of these and all other *A. mysticus* populations was high, being comparatively greater than that recorded between some mainland Australian and Vulnerable Tasmanian populations of the tiger quoll, *Dasyurus maculatus* (Firestone, Houlden, Sherwin, & Geffen, 2000), and at a similar level to that found between populations of *A*.* agilis* with different teat numbers, which may represent incipient species (Appendix S6) (Beckman et al., 2007; Draper, 2012).

Even if the two highly differentiated *A*.* mysticus* populations (Eungella and Cooloola) were excluded, genetic variation was significant and comparable between populations of both *Antechinus* species in southeast Qld. The microsatellite differentiation among these populations in both species was larger than that recorded between populations of the broad‐ranging *A*.* flavipes flavipes* in southeast Australia (Lada et al., 2008) and comparable to same teat number populations of fragmented *A. agilis* (Beckman et al., 2007) (Appendix S6). Indeed, the difference between these southeast Qld populations of each species appears similar to the differentiation within Tasmanian and mainland populations of Endangered, large Australian carnivorous marsupials, which were sampled over a similar geographic range (Cardoso et al., 2009, 2014) (Appendix S6). Unfortunately, few comparative studies have been undertaken on other small carnivorous marsupials, although greater microsatellite differentiation was found between mainland and island populations of the Endangered dibbler, *Parantechinus apicalis* (Mills, Moro, & Spencer, 2004).

In the present study, levels of genetic variation (*AR, H*_e_) were low to moderate for all populations of both species except *A*. *mysticus* at Cooloola, which had much lower variation. These values were comparatively lower than previously reported for other *Antechinus* (Appendix S6). While variation in sample size can influence these values, it is notable that genetic variation in both species was similar to averages reported in the last review of marsupial population genetic diversity (Eldridge, 2010), in which the majority of studied taxa were listed as threatened species. For instance, levels of genetic variation found in the current study were similar to that found for well‐connected populations of two threatened marsupials, the Vulnerable brush‐tailed rock‐wallaby, *Petrogale penicillata* (Hazlitt, Goldizen, & Eldridge, 2006) and mainland populations of the Endangered northern quoll, *Dasyurus hallucatus* (Cardoso et al., 2009). However, genetic variation was higher here than that reported for two other threatened marsupials which occur in well‐connected habitats, the eastern quoll, *Dasyurus viverrinus* (Cardoso et al., 2014) and Tasmanian devil, *Sarcophilus harrisii* (Jones, Paetkau, Geffen, & Moritz, 2004; Appendix S6). In summary, the genetic variation of *A. mysticus* and *A. subtropicus* appears to be within the reported range of some threatened carnivorous marsupials. Our genetic structure analysis also suggests that *A. mysticus* may have experienced a decline and be at high risk of localized extinction at Cooloola (see below).

### 4.2 Genetic differentiation and variation among *A. mysticus* populations

Given its geographic (>700 km) and genetic (>2% at Cytb) isolation from known conspecifics (Baker et al., 2012; Mutton, 2017), it was unsurprising that the Eungella population of *A. mysticus* was strongly differentiated in our study. However, the deep differentiation of Cooloola was unexpected as Cooloola revealed only slight divergence at Cytb (one base pair) from southeast Qld conspecifics and is only 76–155 km from other southeast Qld *A. mysticus* sites (Mutton, 2017). Our observed microsatellite divergence was not the result of misidentification: visual (pelage) inspection (which is diagnostic, see Baker et al., 2012) and Cytb screening prior to population genetic analysis clearly identified all individuals genotyped here as *A*.* mysticus* (Mutton, 2017). Cooloola *A*.* mysticus* also have a similar allele size range to the other *A*. *mysticus* samples genotyped and, like all *A*.* mysticus* in this study, failed to successfully genotype for the locus Aa7Q.

Trap success at Cooloola (0.33%) was an order of magnitude lower than at the other *A. mysticus* sites. This population also exhibited lower genetic diversity (*AR, H*_e_ *, H*_o_) than the other *A*.* mysticus* populations. Taken together, this suggests that the population is both small and isolated, factors which can cause high differentiation in *F*_ST_ values (Weir & Cockerham, 1984). However, few unique and rare alleles were found in the Cooloola population, and the opposite would be expected if the population had long been isolated (Frankham, Briscoe, & Ballou, 2002). In total, these results suggest that the strong differentiation of the Cooloola *A*. *mysticus* from other populations is most likely driven by low abundance and a genetic bottleneck rather than long‐term divergence from conspecifics or incipient speciation. Consequently, the species may be at risk of localized extinction at Cooloola.

However, incipient speciation cannot be completely ruled out, as it has been suggested for *A. agilis* populations which show similarly deep microsatellite, but low Cytb divergence (Beckman et al., 2007; Draper, 2012). However, these putative *A. agilis* species have different numbers of nipples, whereas the nipple number of *A. mysticus* at Cooloola was not different from that recorded for other *A. mysticus* populations. There is also no obvious geographic boundary to vertebrate movement between Cooloola and southern *A. mysticus* populations.

The lack of concordance between microsatellite and Cytb results for the Cooloola *A. mysticus* population may be driven by natural selection acting on Cytb. However, Cytb patterns are otherwise well aligned with known biogeographic barriers (Mutton, 2017) and morphological variation within and between *Antechinus* species (Baker et al., 2015). Furthermore, strong selection pressure on Cytb is often associated with very high altitude environments (Zhang, Lin, Nevo, Yang, & Su, 2013), which was not a factor in the present study. Most likely, the low Cytb and high microsatellite divergence observed within southeast Qld *A*. *mysticus* is primarily driven by the different evolutionary speeds of the two marker types (Sunnucks, 2000). Such an explanation has also been invoked to explain a similar discrepancy between Cytb and microsatellite results in *A. agilis* (Beckman et al., 2007).

### 4.3 Comparative population structure between *A. mysticus* and *A. subtropicus*

It was hypothesized that *A*. *mysticus* utilized a broader and more connected array of habitats in southeast Qld than the relatively high altitude subtropical vine forest and rainforest habitats of *A*. *subtropicus* (Van Dyck et al., 2013). If true, less microsatellite structure between southeast Qld populations of *A*. *mysticus* relative to *A*. *subtropicus* would be expected. However, as indicated above, this was not observed. This disparity seems unlikely to be driven by differing dispersal abilities, as all antechinus appear to share relatively similar life histories and dispersal capacities (Van Dyck et al., 2013). Lower M‐ratio values suggest some of the differentiation in *A*. *mysticus* may be driven by anthropogenic effects (see below). However, given the large degree of differentiation reported, it is also likely that *A*. *mysticus* utilizes a more restricted array of habitats than previously assumed.

*Antechinus flavipes* is the only other *Antechinus* species that occurs throughout southeast Qld. This species is broadly distributed throughout the region but was not caught at any of our study sites. Rather, it is predominantly found in dry, open sclerophyll habitats (Baker & Van Dyck, 2013; Van Dyck et al., 2013). Indeed, *A*. *flavipes* was found in drier habitats nearby a number of sites used in the present study (D'Aguilar, Cooloola, Crohamhurst) (data not shown). It therefore seems likely that the three southeast Qld *Antechinus* species are largely partitioned into separate habitats: *A*. *subtropicus* in closed, high altitude, habitats; *A*. *mysticus* in intermediately closed medium altitude habitats; *A*. *flavipes* in drier, more open, and low altitude habitats. This suggests that *A*. *mysticus*, like *A*. *subtropicus,* is likely isolated to islands of mesic habitat. If so, *A*. *flavipes* might be expected to show relatively less population structure than these species in southeast Qld. While this is yet to be tested in Queensland, *A*. *flavipes* in southeastern Australia is less structured than either of the species in the present study (Lada et al., 2008).

### 4.4 Conservation

M‐ratios were generally much lower in *A*. *mysticus* than *A. subtropicus* (Table 2). This suggests *A*. *mysticus* was previously more connected but has undergone population contractions throughout its range (Garza & Williamson, 2001; Nei, Maruyama, & Chakraborty, 1975). Southeast Qld has experienced a remarkably high rate of land clearing in the 230 years since European colonization (Figure 1). The lower altitude habitats that *A*. *mysticus* favors are also easier to clear and utilize for humans and thus have experienced higher clearing rates (Bradshaw, 2012). This is apparent in two national parks sampled in the present study (Conondale and D'Aguilar) where the high altitude areas in which *A*. *subtropicus* occur are more intact and have been preserved since the 1970s. In comparison, the habitat of *A*. *mysticus* occurs on the edge and outside national parks at both locations, where extensive land clearing has occurred (Figure 1, Bradshaw, 2012) (Table 2).

As well as land clearing, threats such as introduced predators (feral cats, *Felis catus* and foxes, *Vulpes vulpes*) and climate change may have caused population reductions in *A*. *mysticus* and will likely continue to negatively affect both species and many other mammals (Woinarski et al., 2015). Indeed, a recent microsatellite study identified that land clearing and related anthropogenic changes are driving a substantial decline in the population size of southwestern Australian *A. flavipes* (Mijangos, Pacioni, Spencer, Hillyer, & Craig, 2017). However, the evidence of decline throughout the range of *A. mysticus,* which is possibly severe at Cooloola, suggests that *A. mysticus* is at a more immediate extinction risk than *A. subtropicus*. Therefore, we believe *A. mysticus* could warrant threatened species listing. We advocate regular monitoring of *A. mysticus* and prioritization of habitat protection for this species.

Australia has experienced an extraordinarily high rate of mammalian extinction in the last ~200 years (Woinarski et al., 2015). *Antechinus* have previously been considered less at risk of extinction than many Australian mammals in a similar size range. However, the present study and recent listing of four species of *Antechinus* as at threat of extinction suggest this is no longer the case. There is little information on the population structure and abundance of many of Australia's largely endemic marsupial mammals (Woinarski, Burbidge, & Harrison, 2014). In light of the recent declines in *Antechinus* and the ongoing decline of Australian mammals more broadly, it is apparent that the extinction risk of many of these taxa not currently listed as at risk of extinction should now be reassessed.

## CONFLICT OF INTEREST

None declared.

## AUTHOR CONTRIBUTION

TYM, SJF, and AMB designed the study. TYM collected and analyzed the data. DT constructed the maps and provided the vegetation data. TYM wrote the manuscript with assistance from all authors.
