# Supplementary material for: Discovered and disappearing? Conservation genetics of a recently named Australian carnivorous marsupial
Source: Ecol Evol. 2018 Aug 29;8(18):9413–25. doi: 10.1002/ece3.4376 (PMC6194214; doi:10.1002/ece3.4376)
Supplement: Supplementary file 1 [file ECE3-8-9413-s001.docx]

**Appendices**

Appendix 1. Elevation map of south-east Queensland showing the sites sampled in the present study. Circles represent *A*. *mysticus* populations, triangles represent *A*. *subtropicus* populations. The square represents a site at which both species were caught.


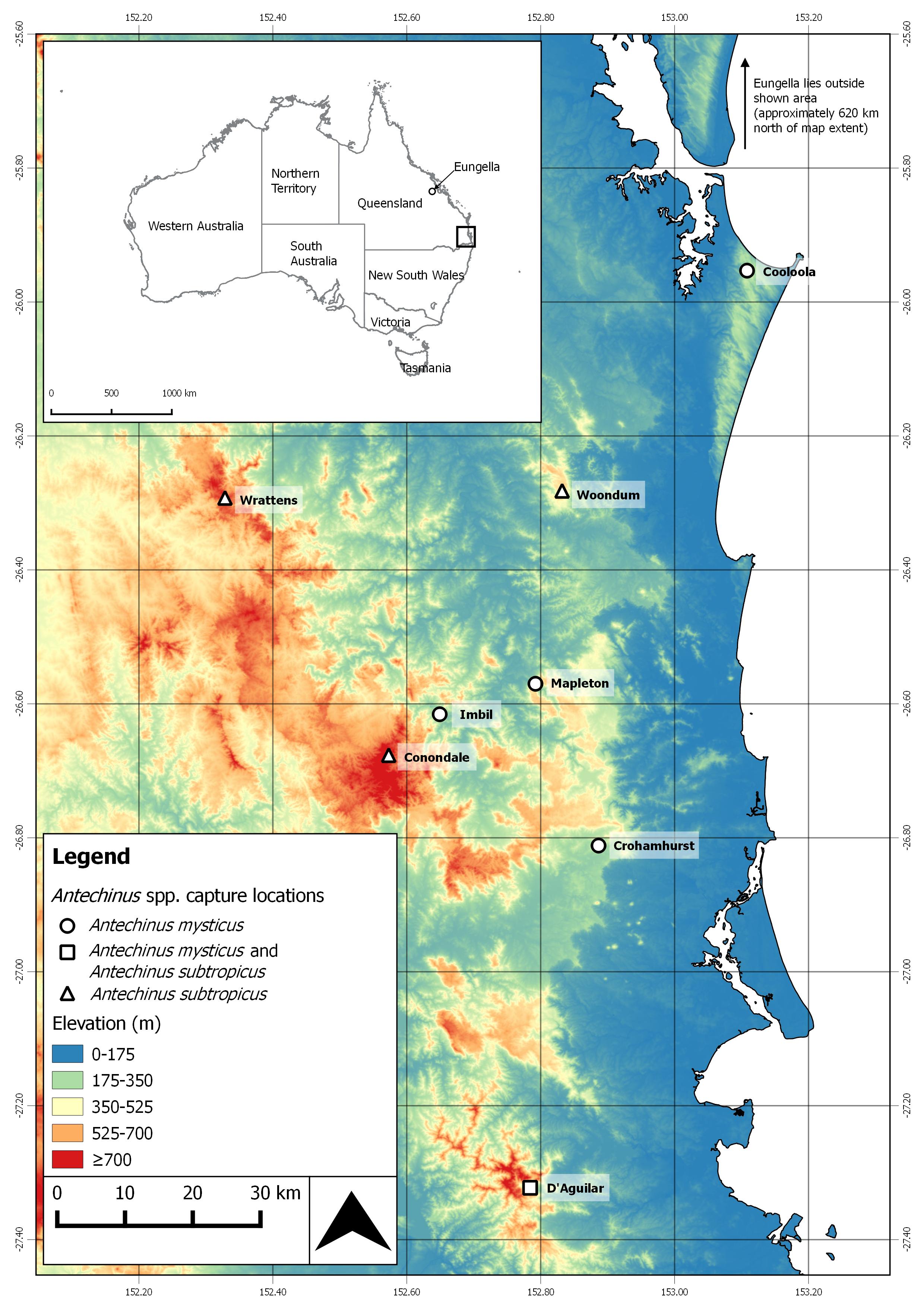


Appendix 2. Geographic location of all sites. ASL = above sea level; *Am* = *A*. *mysticus*; *As = A*. *subtropicus*. Sites are named after the national park in which they occur, except Imbil, which is a state forest and Crohamhurst, a conservation area.

| Name of Site | Latitude and longitude | Metres ASL | *Antechinus* species |
| --- | --- | --- | --- |
| Eungella | -21.16887°S 148.5065°E | 750m | *Am* |
| Cooloola | -25.9532°S 153.1082°E | 140m | *Am* |
| Mapleton | -26.57003°S 152.7922°E | 130m | *Am* |
| Imbil | -26.61553°S 152.6492°E | 150m | *Am* |
| Crohamhurst | -26.81161°S 152.8864°E | 200m | *Am* |
| D’Aguilar | -27.32257°S 152.78423°E | 230-330m | *Am* & *As* |
| Wrattens | -26.29253°S 152.3285°E | 700m | *As* |
| Woondum | -26.28197°S 152.832°E | 460m | *As* |
| Conondale | -26.67661°S 152.573°E | 708m | *As* |

Appendix 3. Regional ecosystems of the study sites. Regional ecosystems descriptions are from the Department of Environment and Heritage Protection, Queensland Government (2009).

| **Name of Site** | **Regional Ecosystem code** | **Regional Ecosystem Description** | **Vegetation structure** |
| --- | --- | --- | --- |
| Eungella |  |  |  |
| Cooloola | 12.2.1 | Notophyll vine forest on parabolic high dunes | Dense |
| Mapleton | 12.12.15a | *Eucalyptus grandis* and/or *E. saligna* tall open forest +/- vine forest understorey, occuring in wet gullies on Mesozoic to Proterozoic igneous rocks. | Mid-dense |
| Imbil | 12.11.10 / 12.11.1 / 12.11.3 | Notophyll vine forest *+/- Araucaria cunninghamii* on metamorphics +/- interbedded volcanics (12.11.10:50%); Simple notophyll vine forest often with abundant *Archontophoenix cunninghamiana* (gully vine forest) on metamorphics +/- interbedded volcanics (12.11.1:45%); *Eucalyptus siderophloia*, *E. propinqua* +/- *E. microcorys*, *Lophostemon confertus, Corymbia intermedia, E. acmenoides* open forest on metamorphics +/- interbedded volcanics (12.11.1:5%). | Dense / dense / mid-dense |
| Crohamhurst | 12.9-10.14 | *Eucalyptus pilularis* tall open forest on sedimentary rocks | Mid-dense |
| D’Aguilar | Clear / 12.12.1 | Simple notophyll vine forest usually with abundant *Archontophoenix cunninghamiana* (gully vine forest) on Mesozoic to Proterozoic igneous rocks | Dense |
| Wrattens | 12.11.10 | Notophyll vine forest +/- *Araucaria cunninghamii* on metamorphics +/- interbedded volcanics | Dense |
| Woondum | Hoop pine plantation bordering 12.12.15a | *Eucalyptus grandis* and/or *E. saligna* tall open forest +/- vine forest understorey, occuring in wet gullies on Mesozoic to Proterozoic igneous rocks (12.12.15a). | Mid-dense |
| Conondale | 12.11.2 | *Eucalyptus saligna* subsp. *saligna* or *E. grandis*, *E. microcorys*, *Lophostemon confertus* tall open forest on metamorphics +/- interbedded volcanics. | Mid-dense |

Appendix 4. Pairwise *D*_EST_ estimates of (a) *A*. *mysticus* and (b) *A*. *subtropicus* populations for 7 and 9 amplified microsatellite loci, respectively. All pairwise comparisons were significantly differentiated (p <0.005).

| (a) | Eungella | Cooloola | Mapleton | Imbil | Crohamhurst |  |
| --- | --- | --- | --- | --- | --- | --- |
| Eungella |  |  |  |  |  | |
| Cooloola | **0.702** |  |  |  |  | |
| Mapleton | **0.595** | **0.452** |  |  |  | |
| Imbil | **0.536** | **0.395** | **0.208** |  |  | |
| Crohamhurst | **0.514** | **0.560** | **0.285** | **0.175** |  | |
| D'Aguilar | **0.449** | **0.467** | **0.257** | **0.192** | **0.291** | |

| (b) | Wrattens | Woondum | Conondale |
| --- | --- | --- | --- |
| Wrattens |  |  |  |
| Woondum | **0.156** |  |  |
| Conondale | **0.283** | **0.194** |  |
| D'Aguilar | **0.397** | **0.327** | **0.327** |

Appendix 5. Graphs of the relationship of ∆*K* to *K* are shown for (a) *A. mysticus* and (b) *A. subtropicus*.

(a)


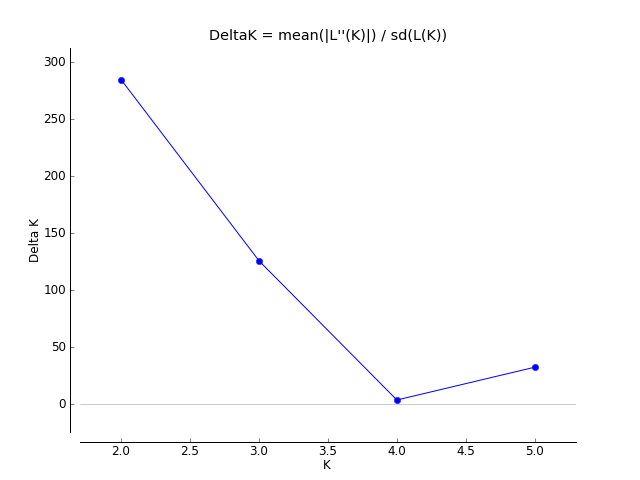


(b)


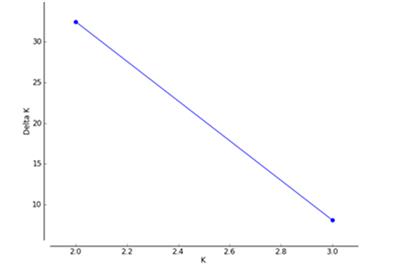


Appendix 6. Genetic diversity estimates for Australian dasyurids. N, sample size; *AR*, allelic richness standardised for allele size; *He*, expected heterozygosity; *Ho*, observed heterozygosity; sample range, geographic range sampled; E, Endangered; V, Vulnerable; NT, Near Threatened; LC, Least Concern; NL, Not Listed; IUCN, The International Union for Conservation of Nature Red List of Threatened Species; EPBC, Environment Projection and Biodiversity Conservation Act List of Threatened Fauna. Translocated or captive populations were not included in the displayed estimations. * indicates that the deeply divergent Wilsons Promontory site was not included.

| Species | References | N | No. of loci | *AR* range (mean) | *He* / *Ho* range (mean) | No. of pop. | Pairwise *F*_ST_ range | Sample range (km) | Conservation listing (IUCN/EPBC) |
| --- | --- | --- | --- | --- | --- | --- | --- | --- | --- |
| Small dasyurids |  |  |  |  |  |  |  |  |  |
| yellow-footed antechinus (*A. f. flavipes*) | Lada et al. (2008) | 744 | 11 | 6.7-7.89 (7.2) | - / 0.76-0.82 (0.79) | 11 | 0-0.107 | ~10-240 | LC/NL |
| agile antechinus (*A. agilis* (6 teat)) | Beckman et al. (2007) | 76 | 9 | 4.8-5.6 (5.3) | 0.66-0.76 (0.71) / - | 4* | 0.017-0.099 | ~20-250 | LC/NL |
| agile antechinus (*A. agilis* (10 teat)) | Beckman et al. (2007) | 96 | 9 | 4.4-6.4 (5.3) | 0.67-0.77 (0.73) / - | 4 | 0.030-0.230 | ~30-600 | LC/NL |
| dibbler (*Parantechinus apicalis*) | Mills et al. (2004) | 57 | 3-7 | - | 0.20-0.73 (0.46) / 0.22-0.75 (0.47) | 3 | 0.27-0.48 | (mainland vs islands) | E/E |
| Large dasyurids |  |  |  |  |  |  |  |  |  |
| northern quoll (*Dasyurus hallucatus*) *-*  islands* | Cardoso et al. (2009) | 39 | 5 | 1.5-2.93 (2.2) | 0.14-0.44 (0.29)/ 0.11-0.35 (0.23) | 2 | 0.666 | - | E/E |
| northern quoll (*Dasyurus hallucatus*) - mainland | Cardoso et al. (2009) | 62 | 5 | 4.59-6.02 (5.2) | 0.58-0.64 (0.61) / 0.56-0.62 (0.58) | 3 | 0.005-0.054 | ~15-120 | E/E |
| tiger quoll (*Dasyurus maculatus*) *-* mainland | Cardoso (2011) | 477 | 6 | - | (-0.583) / - | 19 | 0.139 (mean) | - | NT/V (SE mainland Australia), E (Queensland) |
| tiger quoll (*Dasyurus maculatus*) - mainland | Firestone et al. (2000) | 69 | 6 | 3.6 (mean) | (~0.6) / - | 7 | 0.000-0.429 | 15-1600 | NT/V (SE mainland Australia), E (Queensland) |
| tiger quoll (*Dasyurus maculatus*) - Tasmania | Firestone et al. (2000) | 11 | 6 | 4.05 (mean) | (~0.6) / - | 1 | 0.25-0.97 (from mainland *D. masculatus*) | - | NT/V |
| eastern quoll (*Dasyurus viverrinus*) – mainland | Firestone et al. (2000)) | 38 | 6 | 4.9 (mean) | (~0.7) / - | 2 | - | - | Extinct |
| eastern quoll (*Dasyurus viverrinus*) – Tasmania | Cardoso et al. (2014) | 425 | 7 | 1.86-3.97 (3.2) | 0.31-0.56 (0.46) / 0.31-0.56 (0.46) | 10 | (0.131) | ~25-280 | E/E |
| eastern quoll (*Dasyurus viverrinus*) – Tasmania | Firestone et al. (2000) | 93 | 6 | 4.0 (mean) | (~0.55) / - | 3 | 0.078-0.222 | ~120-300 | E/E |
| tasmanian devil (*Sarcophilus harrisii*) – Tasmania | Jones et al. (2004) | 262 | 11 | - | 0.39-0.47 (0.42) / 0.40-0.48 (0.42) | 6 | 0-0.1895 | 10-340 | E/V |
